# Age-Related Difficulty of Listening Effort in Elderly

**DOI:** 10.3390/ijerph18168845

**Published:** 2021-08-22

**Authors:** Chanbeom Kwak, Woojae Han

**Affiliations:** 1 Laboratory of Hearing and Technology, Research Institute of Audiology and Speech Pathology, College of Natural Sciences, Hallym University, Chuncheon 24252, Korea; cksqja654@gmail.com; 2 Division of Speech Pathology and Audiology, College of Natural Sciences, Hallym University, Chuncheon 24252, Korea

**Keywords:** aging, listening effort, aged hearing, listening environment, direction

## Abstract

The present study identifies the combined effects of aging and listening environment related factors, such as directionality, types of stimuli, and the presence of background noise. A total of 50 listeners with normal hearing (25 older adults and 25 young adults) participated in a series of tasks. The detection task using tone and speech and a speech segregation task with two levels of background noise were conducted while sound was randomly presented via eight directional speakers. After completing each task, a subjective questionnaire using a seven-point Likert scale was asked to measure the amount of the subjects’ listening effort in terms of speech, spatial, and hearing quality. As expected, the amount of listening effort required in all the experiments for the older group was significantly higher than for their young counterparts. The effects of aging and types of stimuli (tone and speech) also showed different patterns of listening effort for the older adults and younger adults. The combined interaction of aging, directionality, and presence of background noise led to a significantly different amount of listening effort for the older group (90.1%) compared to the younger group (53.1%), even in the same listening situation. These current results, when summarized, indicated weak tone detection ability at high frequencies occurred in the elderly population but the elderly could improve their ability by using speech sounds with broad-band spectrum energy. We suggest that a warning signal when using speech rather than a single tone is more advantageous for the elderly in a public environment. It is also better to converse with the elderly by avoiding situations where noise from behind can interrupt.

## 1. Introduction

Aging populations are not a problem limited to only one country, but instead a global issue. Thus, the health problems of the elderly should be addressed at the national public health level, rather than as an individual problem.

Elderly over the age of 65 often experience several difficulties in listening environments due to their decreased ability to communicate [1,2,3], which eventually leads to reduced social connections and less quality of life [4,5]. At the same time, the elderly can have problems with situational awareness of daily life [6] including a fire alarm, car horn in traffic, oncoming objects on street, and falling objects [7,8]. These age-related issues often accompany age-related hearing loss (ARHL or presbycusis), which is one of the three major chronic elderly diseases [5] and a secondary cause of social, functional, and psychological atrophy [9].

With aging, both the peripheral and central auditory systems gradually lose their functions [1,2,3,10]. Thus, the elderly often shows poor performance in their orientation sense to multiple sound sources and find it hard understand speech in the presence of background noise [3,10,11], also resulting in the aforementioned issues of the elderly. Dobreva and colleagues posited that older adults affected by aging of their auditory systems had much poorer performance in sound localization tasking than did younger adults [10]. Even when the researchers controlled for the aging factor in the peripheral auditory system (i.e., hearing thresholds in the high-frequency range), the elderly showed lower accuracy and/or precision in the sound localization task. This issue could explain that even the peripheral degeneration of the auditory system controlled and/or compensated for using acoustical features (i.e., frequency of stimuli), such aging negatively affects and deteriorates the central auditory system [10]. Aging continues to negatively affect cognitive aspects as well. When the elderly population is struggling to localize and understand incoming sounds, they spend more cognitive resources to listening well, and such exertion consequently causes more fatigue [12]. This cognitive heavy work is defined as listening effort (or mental effort) for the listeners [12,13].

There are certain important underlying mechanisms of cognitive processing (i.e., memory, comprehension, attention, and speed of processing). These mechanisms are easily affected by aging. As a result, there is not only a decreased performance of task associated with memory, comprehension, and attention; there is also delayed speed processing identification for the elderly [14]. Pichora-Fuller and Singh confirmed that cognitive processing dramatically increased when the elderly was faced with difficult listening environments (i.e., presence of background or surrounding multiple sound source to focus) and/or challenging inherent characteristics due to aging [15]. This finding was also supported by a study by Kwak et al., when the elderly was placed in the same listening environments, such as church and a senior welfare center, there was an increased amount of listening effort expended than that of young listeners [3]. That is, if the older adults try to overcome their difficulties and seek higher auditory performance in difficult listening environments, their cognitive resources become exhausted, and they more easily will avoid listening situations. Thus, the great burdened physical and/or cognitive listening for the elderly should be considered based on the scientific data.

Although various contemporary research efforts have focused on higher and/or improved performance for the elderly, their cognitive burden expressed as the listening effort could not be considered. We believe that the results of the present study will serve as a positive opportunity to better understand the listening characteristics and required effort of the elderly. In light of this goal, the present study investigated the combined effects of aging, different directionality, types of stimuli, and presence of background noise on listening effort. As the hypothesis, factors related to the challengeable listening environments such as various directionality especially opposite (diagonal) side, differences between stimuli in terms of meaning and/or contextual cue of itself, and presence of background noise were negatively related to the listening effort in simulated real-listening situations, when considering the relationship between aging and expected listening effort for understanding.

## 2. Materials and Methods

### 2.1. Participants

Considering the need for minimum of 48 subjects based on the sample size calculation of the G-Power program [16], it was decided to have 50 native Korean speakers with normal hearing thresholds participated in this study. Half of them (9 males and 16 females) were designated for the older group (OG), who averaged 73.20 ± 4.80 years in age. The other 25 subjects (13 males and 12 females) were included as the young control group (YG; mean age: 22.92 ± 1.66 years old).

The hearing screening tests were conducted before starting the experiments. Having no negative otological history, all the participants also had a normal middle ear function (i.e., A-type of tympanometry). Their hearing sensitivity fell within the normal range for the testing frequencies between 250 and 4000 Hz as a function of age [17]. In addition, the Korean version of the mini-mental state examination (MMSE) was applied to screen the normal cognitive ability of the participants based on 25 or higher scores [18]. The results of these screening tests for two groups are summarized below in Table 1.

The procedures were approved by the Institutional Review Board of Hallym University (#HIRB-2019-063) and the experiments were conducted in compliance with the Declaration of Helsinki, International Conference of Harmonisation Guidelines for Good Clinical Practice.

### 2.2. Simulation Setting

**Figure 1:**
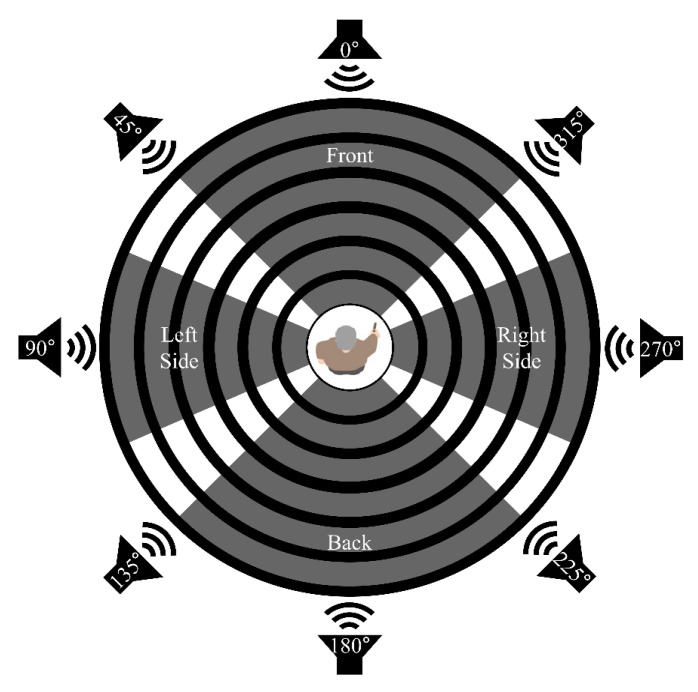
Diagram of the eight speakers’ measurements of the listening effort as a function of direction.

In a sound-proof booth, eight directional speakers (Yamaha HS5, Hamamatsu, Shizuoka, Japan) were set to perform a series of experiments. Figure 1 describes the simulation setting where the subject sitting in the center of the chamber was 1 meter away from each speaker; that is, speakers were spatially separated at 45° intervals. For example, the first speaker was aligned to the subject (0°) and the other speakers were located at 45°, 90°, 135°, 180°, 225°, 270°, and 315°, respectively, in a counterclockwise direction. Furthermore, to make participants easy to identify, each speaker was assigned a number (i.e., 1 to 8) in the same manner as the degree of speakers.

### 2.3. Measurement Tools

#### 2.3.1. Listening Effort

A subjective questionnaire using a 7-point Likert scale was gathered to evaluate listening effort [19]. The questionnaire asked the subject to report the amount of effort when listening to the stimuli, from strongly effortless (1 point) to strongly effortful (7 points) for each experimental condition (See Appendix A for the questionnaire).

#### 2.3.2. Hearing Evaluation

Both simple tone and meaningful speech stimuli were used. For the simple tone, four types consisting of two low frequencies (i.e., 250 and 500 Hz) and two high frequencies (i.e., 4000 and 8000 Hz) simulated the listening situation. Each tone was presented five times per a speaker, for a total of 160 times (8 speakers × 4 different frequencies × 5 tones). For the meaningful speech stimuli, the Korean Hearing in Noise Test (K-HINT) mixed with target sentences and babble noise with 12 talkers was used [20]. The conditions were repeated 40 times (8 speakers × 5 sentences) under two different background noise levels (e.g., 0 dB and –6 dB signal-to-noise ratio). Each stimulus was pseudo-randomly presented at each individual’s the most comfortable level (MCL), which expressed individual’s sensation level and had been obtained during the hearing screening stage. As all of the experiments were conducted in the sound field situation, all patients were fixed their head to minimize the localization cue such as interaural time and/or level differences.

#### 2.3.3. Experimental Procedures

The subjects were instructed to fix their heads with no movement when the stimuli presented. As head movement or head turning can affect the ability of localization, which then was compensated for by the auditory cues, such as interaural time, level, and phase difference [10], the head movement or turn of participants had to be controlled.

All participants were asked to listen to stimuli being presented from the speaker and then report the speaker number they thought had generated that sound. The responses were regarded as correct when the number of the speaker with the stimulus matched the number of the speaker that answered to the subject under the tone presentation. On the other hand, when listening to the speech (or sentences) under background noise from various directions, the participants repeated what they heard. The responses were transformed in terms of accuracy (i.e., correct percentage) with no contextual and semantic errors occurring. Finally, the amount of listening effort was measured subjectively immediately following each experiment.

#### 2.3.4. Data Analysis

A statistical analysis was carried out using SPSS software (IBM Corporation, Armonk, NY, USA). A paired *t*-test and an independent *t*-test were conducted to compare the results of the listening effort in two groups for the conditions. To identify the main effect and interaction of the accuracy for each experiment and the self-reported listening effort between the two groups, a mixed two-way analysis of variance (ANOVA) was applied. As necessary, Bonferroni correction was used for multiple comparisons.

In addition, a Pearson correlation coefficient and stepwise multiple linear regression analysis were conducted to confirm any effect of various variables for listening effort. As the first step, all data were divided into inherent and external variables. Inherent variables included hearing thresholds, gender, age, and the results for the K-SSQ questionnaire [21]. External variables contained directions of signal, frequency of tone signal, and level of background noise. As multiple variables that could influence the listening effort existed [3,10,12,15,19,22], this kind of data sorting could control for the complex interactions between the variables and provide clearer and more straightforward outcomes. After the data sorting, the Pearson correlation coefficient and a stepwise multiple linear regression analysis were applied to both the inherent and external variables to see whether certain variables might influence the listening effort. The statistically significant criterion was *p* < 0.05.

## 3. Results

### 3.1. Comparison of the Amount of Listening Effort between the Groups

**Figure 2:**
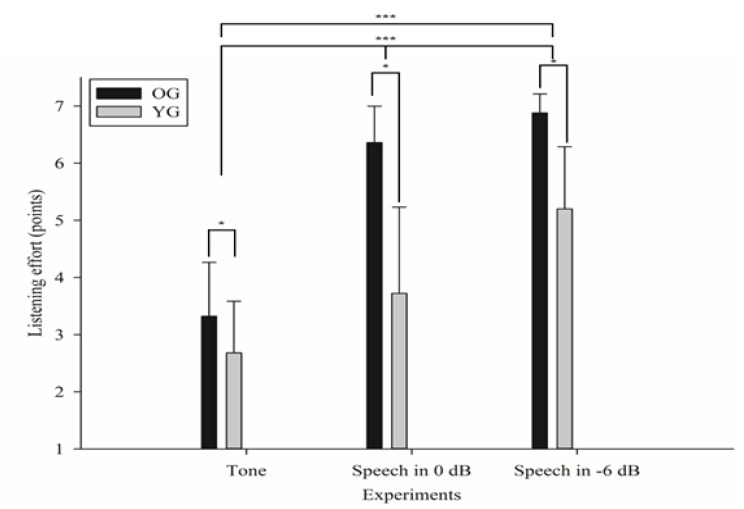
Group comparisons for the amount of listening effort for the conditions. *, *p* < 0.05; ***, *p* < 0.001.

In Figure 2, the OG shows 3.32 points (SD: 0.95), 6.36 points (SD: 0.64), and 6.88 points (SD: 0.33) for tone, speech in 0 dB SNR, and speech in –6 dB SNR conditions, respectively. For the meaningless tone in a quiet environment, little listening effort was required compared to a lot of effort required when presenting a noise and meaningful speech. As the control group, the YG reported the amount of listening effort as 2.68 points (SD: 0.75), 3.72 points (SD: 0.79), and 5.20 points (SD: 0.91) for the three conditions, suggesting that YG used much less effort than the OG. 

The results of a mixed two-way ANOVA with repeated measures confirmed a significant main effect in age (F(1,48) = 139.044, *p* < 0.001, η^2^ = 0.743) and conditions (F(1.349,64.744) = 245.509, *p* < 0.001, η^2^ = 0.836). The interaction between age and condition (F(1.349,64.744) = 25.582, *p* < 0.001, η^2^ = 0.348) was also significant. The overall results showed that the OG (mean: 4.80 points, SD: 1.96) needed significantly higher listening effort than did the YG (mean: 3.36 points, SD: 1.48). To consider the significance of interaction between age and condition, however, the OG already had demonstrated a lot of listening effort even under the 0 dB SNR condition, so only a difference of 0.52 points was found between the 0 dB SNR and −6 dB SNR conditions. Nevertheless, the YG required a listening effort of only 3.72 points at 0 dB SNR, showing a step-up in listening effort depending on the difficulty of listening.

The results in listening effort among the conditions which were analyzed using an independent *t*-test demonstrated that OG showed a significantly higher amount of listening effort in speech in the −6 dB SNR (t = −8.649, *p* < 0.001), 0 dB SNR (t = −12.985, *p* < 0.001), and tone (t = 2.654, *p* = 0.011) conditions than did YG. In addition, the results of a paired *t*-test which compared the amount of listening effort for three conditions showed that speech in the −6 dB condition required more effort than speech in the 0 dB condition (*t* = −9.707, *p* < 0.001) and tone condition (*t* = −17.502, *p* < 0.001). Additionally, speech in the 0 dB condition showed a significantly higher amount of listening effort than did tone condition (t = −9.379, *p* < 0.001).

### 3.2. The Correlation between Listening Effort and Variables

#### 3.2.1. Inherent Variables That Affected Listening Effort

There was a significantly strong correlation between listening effort and inherent variables in speech segregation from the noise task. Table 2 summarizes the results for listening effort required to each task and inherent variables of all participants in terms of correlation coefficient and its significance values.

In detail, the age variable significantly and positively correlated with the tone detection task (r = 0.286, *p* = 0.044) and speech information segregation from noise in both 0 dB SNR (r = 0.856, *p* < 0.001) and −6 dB SNR (r = 0.762, *p* < 0.001) environments. Additionally, listening effort and hearing thresholds were significantly correlated with the speech segregation from noise task of 0 dB and −6 dB SNR although the hearing thresholds were not a key variable listening effort needed to detect tones presented from the various directions.

#### 3.2.2. External Variables Affecting Listening Effort

Table 3 summarizes the results for the Pearson correlation coefficient between listening effort and external variables. 

Overall, OG showed a more negative and stronger correlation between the experimental stimuli and the directions compared to the results for YG. For tone detection, OG had significant correlation between listening effort and frequency of the presented tones in the higher frequencies (i.e., 4000 and 8000 Hz), but not in the low frequencies (i.e., 250 and 500 Hz) except for 270° (r = −0.456, *p* = 0.022) and 315° (r = −0.546, *p* = 0.005) in 500 Hz. YG showed several significant and negative correlations in 315° at 250 Hz (r = −0.412, *p* = 0.041) and 4000 Hz (r = −0.497, *p* = 0.012) and 0° (r = −0.415, *p* = 0.039) at 500 Hz. Interestingly, while OG showed a significant correlation in the 0 dB SNR condition of speech segregation from noise task, YG showed a significant correlation in the −6 dB SNR condition. OG demonstrated a significant correlation in 0° (r = −0.479, *p* = 0.015), 180° (r = 0.482, *p* = 0.015), and 270° (r = −0.481, *p* = 0.015) of the 0 dB SNR condition. Otherwise, YG showed a significant correlation in 45° (r = −0.578, *p* = 0.002), 180° (r = −0.633, *p* = 0.001), and 315° (r = −0.410, *p* = 0.042) of −6 dB SNR condition.

### 3.3. Multiple Regression Model for Listening Effort

#### 3.3.1. Listening Effort for Tone Detection

To identify the relationship between listening effort and other possible factors (i.e., hearing thresholds, and directionality) in tone detection, a stepwise multiple linear regression analysis was conducted. The dependent variable was the amount of listening effort for each group. The equation for the listening effort with tone detection in OG was followed:

ŷ = 3.842 − 0.684 ∗ 0° at 4000 Hz − 0.516 ∗ 250 Hz of HT + 0.487 ∗ 1000 Hz of HT + 0.285 ∗ 0° at 500 Hz

The Equation for the listening effort with tone detection in YG was followed:

ŷ = 5.348 − 0.497 ∗ 315° at 4000 Hz

Table 4 shows the results of multiple regression analysis for both groups. The results for the multiple linear regression model of OG showed 0.826 of adjusted R2 (*p* < 0.05). Specifically, 0° with 4000 Hz pure-tone (β = −0.684, *p* < 0.001) was the most significant predictive variable for the listening effort. Hearing thresholds of 250 Hz (β = −0.516, *p* < 0.001) and 1000 Hz (β = 0.487, *p* < 0.001), and 0° with 500 Hz (β = 0.285, *p* < 0.05) were followed. The results of a stepwise multiple linear regression model of YG showed relatively low adjusted R2 (0.214, *p* < 0.05) compared to that for OG. Similar to the results of OG, 315° with a tone of 4000 Hz (β = −0.497, *p* < 0.05) was a significantly predictive variable for the listening effort.

#### 3.3.2. Listening Effort and Speech Segregation from Noise

With consistent purpose, stepwise multiple linear regression analysis of speech segregation from noise was carried out. The equation for speech segregation from 0 dB noise in OG was followed:

ŷ = 5.690 + 0.402 ∗ 180° − 0.507 ∗ 360°

The equation for speech segregation from −6 dB noise in OG was followed:

ŷ = 7.802 + 0.621 ∗ 250 Hz of HT − 0.530 ∗ 0°

The equation for speech segregation from 0 dB noise in YG was followed:

ŷ = 4.084 − 0.423 ∗ 3000 Hz of HT

The equation for speech segregation from −6 dB noise in YG was followed:

ŷ = 11.261 − 0.552 ∗ 180° − 0.354 ∗ 3000 Hz of HT − 0.380 ∗ 45°

Based on Table 5, the multiple linear regression model of OG in 0 dB SNR indicated that the adjusted R2 was 0.440 (*p* < 0.05). In the regression equation, the 180° direction (β = 0.402, *p* < 0.05) was a more significantly predictive variable than the 360° direction (β = −507, *p* < 0.05) in OG. In the case of the −6 dB SNR condition, the model for multiple linear regression showed 0.384 of adjusted R2 (*p* < 0.001) for OG.

On the other hand, YG showed a relatively lower coefficient of determinants (adjusted R2 = 0.143, *p* < 0.05) than did that of OG in the 0 dB SNR condition. In the regression equation, a hearing threshold of 3000 Hz (β = −0.423, *p* < 0.05) was the only significant predictive variable in 0 dB SNR condition. Interestingly, −6 dB SNR condition showed higher adjusted R2 (0.562, *p* < 0.05) than did the 0 dB SNR condition. The 180° of direction (β = −0.552, *p* < 0.001) was the most significant predictive variable in the YG regression equation. A hearing threshold of 3000 Hz (β = −0.354, *p* < 0.05) and 45° of direction (β = −0.380, *p* < 0.05) were followed.

**Figure 3:**
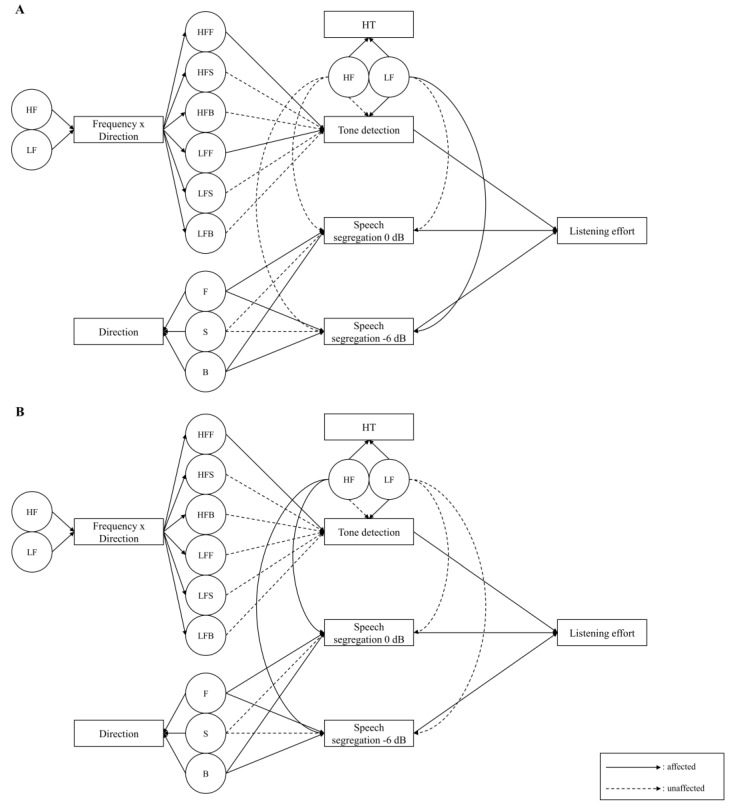
Multiple linear regression model of the older adults (**A**) and young adults (**B**). HF, high frequency; LF, low frequency; HFF, high frequency front; HFS, high frequency side; HFB, high frequency back; LFF, low frequency front; LFS, low frequency side; LFB, low frequency back; F, front; S, side; B, back; HT, hearing threshold.

The overall results for all variables and listening efforts are depicted in Figure 3. Based on the aging effect, the OG and YG each showed the process of listening effort measurement systematically. In panel A, the OG was only influenced by the front direction in the tone detection task. Additionally, the directions of front and back significantly affected each condition of speech segregation from the noise task. Interestingly, the hearing threshold of low frequency was one of the inherent variables significantly influenced on −6 dB SNR condition in speech segregation from the noise task. Unlikely for the OG, the results of the YG in panel B showed that the front direction of high-frequency stimuli affected the tone detection task. However, the effect of directions in speech segregation from a noise task was the same with the OG. For the effect of the hearing threshold for speech segregation from the noise task, only the high frequency of the hearing threshold showed significance for both conditions.

## 4. Discussion

The current study confirmed the combined effects of aging, different directionality, types of stimuli, and presence of background noise on listening effort. The results of current study thus provide comprehensive information about the elderly, especially for the listening behaviors toward stimuli and environments.

### 4.1. Did the Older Adults Spend More Listening Effort than the Young Adults to Recognize Incoming Signals from Various Directions?

We found that listening ability for tone and speech coming from different directions with and without background noise can be negatively affected by aging, with these results being directly or partially supported by previous studies [3,6,10]. Furthermore, aging resulted in a larger amount of listening effort for the elderly than their younger counterparts [3], even in equal listening situations, because of the working of both the peripheral and central auditory and cognitive systems [10]. Although both OG and YG showed increased listening effort as the level of listening condition became progressively harder, the pattern of saturation point of listening effort differed due to aging. The OG showed approximately twice the listening effort (6.36 points) at speech segregation from a background noise of 0 dB than tone detection (3.32 points) and maintained a similar amount of listening effort at a 6 dB condition of speech segregation from background noise. However, the YG that was not affected by the aging showed a large increase in listening effort at speech segregation from a background noise of −6 dB (5.20 points) than for the other listening condition of tone detection (2.68 points) and 0 dB of speech segregation from background noise (3.72 points). That is, the amount of listening effort of the OG was significantly affected by the types of stimuli (tone to speech), which was unlike the YG (speech to speech, but a different noise level). The hierarchy of auditory skills was defined following four levels: detection, discrimination, identification, and comprehension [23]; the tone detection task and speech segregation from the noise task were clearly differentiated. While the tone detection task was classified into a detection level, which defined the awareness of the presence or absence of sound, speech segregation from the noise task clustered the comprehension level, which referred to the highest of the four levels of auditory skills and connected the auditory perception with cognitive and/or language abilities [24]. The tone detection task required detection of the pure tone in various directions and was a basic level of auditory perception. 

However, in the case of speech segregation from noise, the task required listening to and repeating the sentence generated in multiple sound sources, and this task was considered as a complicated auditory perception skill. In addition, the listening effort was made up of a complex construct, such as auditory perception, attention, and cognitive resources. To compare the detection and the comprehension levels, the comprehension level of auditory perception obviously required more listening effort than the detection level, even in normal adults. Along with the aging effects, the central auditory and cognitive processing ability of the OG deteriorated, which decreased the ability of both auditory and cognitive domains and might have had more influence on the comprehension level, but not the detection level.

### 4.2. Are Specific Internal and External Variables Affecting Listening Effort Due to Aging?

None of the inherent variables, except for age, were significant in the tone detection task. However, the results of the correlation between speech segregation from two background noise and inherent variables demonstrated that all the variables except for gender significantly and positively correlated with listening effort. Moreover, the correlation coefficients increased along with the level of the listening situations. That is, they differentiated from 0.286 (tone detection) to 0.856 (0 dB of speech segregation from noise) to 0.762 (−6 dB of speech segregation from noise) relative to listening effort. To differentiate the effects of the different types of stimuli, tone stimuli had significantly positive, but weak, correlation coefficients with listening effort. However, the correlation coefficients changed not only statistically significantly and positively, but also delivered a strong correlation between listening effort and speech signal. These relationships between listening effort and aging in the present study are supported by the previous study by Rail et al. [25]. They posited that the aging auditory systems occurred due to complex interactions with increasing hearing thresholds decreased the spectral and temporal processing ability and neural degeneration in both outer and inner hair cells [25], which are the underlying mechanisms for auditory signal processing and sound localization. That is, a degraded structure and function of the auditory system due to aging showed a deteriorated ability for signal processing, such as tone and speech; this negative effect of aging then led to an increased amount of listening effort. 

In view of the external variables, the listening effort for high-frequency tones were more negatively correlated with direction, which were relative to front (0°, 45°, and 315°) and sides (90° and 270°) in the tone detection task of the OG. However, only two directions, 270° and 315°, negatively correlated with listening effort for low frequency tones. As expected, the YG showed a decreased number of directions, which correlated with listening effort. Listening effort for both low and high frequencies showed a correlation between relatively front (0° and 315°) direction. To differentiate the age group in an external variable analysis, OG showed negative correlation with 0° and 270° and positive correlation with 180° in only the 0 dB SNR condition. Unlikely for OG, the results of YG showed a negative correlation with 45°, 180°, and 315° in only the −6 dB SNR condition. 

These results may stem from the negative effects of aging on directionality with the same meaning as sound localization. In detail, decreased hearing thresholds in the high-frequency region was due to age-related hearing loss and the characteristics of signal influences on sound localization, especially in any front-back confusion in the horizontal plane [10,26,27,28]. Abel and colleagues found that older adults showed poor sound localization ability for both accuracy and error rate in various directions [26]. They posited that the central auditory dysfunction upon aging negatively affected sound localization and decreased spectral information processing ability and could be a key contributor in front–back confusion. These results suggest that sound localization of nonspeech sounds obviously is affected by a deteriorating central auditory system due to aging. 

Shinn-Cunningham and Best also identified sound localization in speech sounds while indicating that even though the level of speech signal is sufficient to perceive, the information of ultra-high frequency (i.e., above 8 kHz) in a speech signal is important for sound localization. Thus, despite a declining peripheral auditory system compensated by a sufficient presentation level, the process of auditory streaming and segregation which are the determinants of sound localization can be damaged by the interaction of an aging peripheral and central auditory system [29].

The results of the multiple regression analysis were confirmed, and there were interesting findings for the multiple regression of speech segregation task and listening effort. When background noise added the speech signal was presented, the elderly showed poorer performance in speech recognition ability than the YG for all background noise conditions. As expected, the accuracy of speech perception was significantly decreased when the level of noise increased from 0 to −6 dB SNR. Furthermore, it is well known that the listening process accompanies both auditory and cognitive aspects, especially attention [14,29,30] and this listening process, especially listening, in the presence of a background decline with aging. Based on the results of the multiple linear regression model in the current study, OG showed that front (0°) and back (180°) were key contributors to listening effort.

In contrast, in the case of −6 dB SNR, 0° direction, which as background noise was not presented, was a predictive variable of listening effort. In the case of YG, 180° direction in −6 dB SNR was the only contributing factor to listening effort. Even though the front–back confusion was not the intended purpose of the current study, OG showed 180° and 0° as predictable variables in the 0 dB SNR condition. Similarly, with OG, the results of YG also demonstrated that 180° was the highest regression coefficient variable in the −6 dB SNR condition. In addition, while the other variables as mentioned above showed negative correlation and regression coefficients, only 180° of the 0 dB SNR condition in OG was a positive value. These results may explain that 0° of the 0 dB SNR condition in OG and 180° of the −6 dB SNR condition of YG suggest that both OG and YG had to spend more effort to understand the speech signal and discriminate directions when signal was presented. However, the accuracy of speech segregation performance in 180° of 0 dB SNR condition increased, and the amount of listening effort increased. This result suggests that even when OG had high accuracy in 180° of 0 dB SNR condition, the elderly confused the directions of front and back, and this auditory and cognitive confusion was expressed as listening effort. Shinn-Cunningham and Best argued that an impaired peripheral auditory system due to aging impeded the formation of auditory signals and caused cognitive overload [29]. Additionally, Kwak and his colleagues reported that if older adults showed a similar level of performance in a challenging condition (i.e., presence of background noise and reverberation) with their younger counterparts, the amount of listening effort is significantly higher in the older adults [3]. These results show that even when the older adults overcame the difficulties and showed a higher auditory performance in difficult listening environments, their cognitive resources were already exhausted. In other words, the elderly, influenced by aging, found it difficult to segregate auditory information in one sound source, and this difficulty could increase as the number of sound sources increased.

### 4.3. Limitations of Study and Future Directions 

Although the current study identified the aging effects for the OG and YG that met the criteria of normal hearing with age-specific hearing thresholds [17] and cognitive functions, it is necessary to compare these results to the negative effects of natural aging for cognitive functions, such as mild cognitive impairment for the same age group. The obvious gap of hearing thresholds between two groups could be confounders and this issue may have affected the results of the current study. In this way, a more precise aging effect on cognitive functions can be identified. Additionally, the benefits from directional hearing aids can be examined to enhance the decreased ability of sound localization and compensate the cognitive burden for older adults in future studies. Since the elderly had poor sound localization ability and speech recognition ability in noise and expended a higher listening effort, we believe that hearing aids the use of directionality and noise reduction technology may play an important role to compensate their declined auditory and cognitive functions. Hornsby investigated the relationship between hearing aid use and listening effort for the hard-of-hearing [31]. Using a dual-task paradigm that consisted of word recall and reaction time, he posited that the usage of hearing aids alleviates the amount of listening effort and/or mental fatigue for the hard-of-hearing. Although Hornsby’s study did not identify the effects of directionality for hearing aid on listening effort and/or mental fatigue, there is a distinct possibility that a directional function of hearing aid could be very effective in reducing listening effort and/or mental fatigue. Thus, we sought to describe any positive and side effects of directional hearing aids in the elderly. These hearing aids can increase the audibility of direction and reduce the amount of listening effort [12] and thus improve elderly communication skills in everyday life and for situational awareness [6], such as car horns in traffic, oncoming objects on the street, and falling objects [7]. It is important to protect the safety and health of the elderly by providing better guidelines (i.e., warning alarms presented at the front direction) based on the scientific data on their listening ability from multiple directions.

It is also important to consider the gap between a laboratory setting and real listening situations. The results of the current study clearly demonstrate that the OG showed their maximum amount of listening effort in 0 dB SNR condition and gave up in the −6 dB SNR condition to speech perception from various directions. Wu and colleagues reported that if a person becomes cognitively overloaded due to external variables (i.e., background noise) during a listening task, they will give up on that task [11]. They measured response times for speech recognition tasks in various SNR conditions for both older adults with hearing loss and normal young adults. The results offered by Wu and colleagues demonstrated that both old and young adults showed a shorter response time in specific and challengeable SNR conditions such as −2 dB. In real listening situations, this same giving up point (i.e., −2 dB SNR) can become clustered by “Conversation at the checkout counter or with friends while shopping” or “Conversation on a train, in the station hall, or close to the train platform” or “Conversation in a moving car”, which ranged from 0 to –5 dB SNR [32]. Finally, if considering that females respond differently in any hearing condition, an equal number of participants in each gender group should be included for the following study. 

## 5. Conclusions

The present results clearly suggest that the combined effects of aging, directionality, types of auditory signal, and presence of background noise negatively influences listening effort; this effect was dominant in both front and back directions. The older group also required much more listening effort than their younger counterparts, even in equal listening conditions. We thus concluded that older people have weak tone detection ability in high frequencies but can improve that ability by using speech sounds with broad-band spectrum energy. To address the presence of background noise and multi-directions in real listening situations, the presence and levels of background noise and their various directions (especially front and back) should be considered to provide more comfortable and effortless communication behavior for all older adults.

## Figures and Tables

**Table 1:** A comparison of participant group mean and standard deviation for hearing sensitivity and MMSE.

Group	Age (Years)	Hearing Thresholds (dB HL) in Frequency						MMSE (Score)
		250 Hz	500 Hz	1000 Hz	2000 Hz	4000 Hz	8000 Hz	
OG	73.05	16.90 ± 6.41	16.80 ± 6.69	16.50 ± 8.58	17.20 ± 8.61	26.50 ± 15.32	45.30 ± 16.60	27.04 ± 1.14
YG	22.92	2.90 ± 0.41	2.30 ± 0.25	1.70 ± 0.16	2.20 ± 0.34	4.70 ± 0.10	4.80 ± 0.72	29.13 ± 0.51

Abbreviations: OG, older group; YG, young group; MMSE, mini-mental state examination.

**Table 2:** Results of the Pearson correlation for listening effort and inherent variables for three conditions.

Stimuli	Age	Gender	Hearing Thresholds (dB HL) in Frequency							
			250 Hz	500 Hz	1000 Hz	2000 Hz	3000 Hz	4000 Hz	6000 Hz	8000 Hz
Tone	0.286 *	−0.034	0.090	0.201	0.358 *	0.388 **	0.325 *	0.299 *	0.259	0.263
0 dB SNR	0.856 **	0.076	0.703 **	0.673 **	0.663 **	0.640 **	0.665 **	0.610 **	0.735 **	0.781 **
−6 dB SNR	0.762 **	0.168	0.629 **	0.594 **	0.585 **	0.567 **	0.610 **	0.553 **	0.651 **	0.695 **

Abbreviations: SNR, signal-to-noise ratio; *, *p* < 0.05; **, *p* < 0.01.

**Table 3:** Results of the Pearson correlation between listening effort and external variables for the two groups.

	Types of Stimuli		Directions of Speaker							
			0°	45°	90°	135°	180°	225°	270°	315°
OG	Tone	250 Hz	0.287	0.084	−0.307	−0.211	−0.072	−0.381	−0.331	−0.354
		500 Hz	−0.172	−0.103	−0.383	−0.190	−0.314	−0.102	−0.456 *	−0.546 **
		4000 Hz	−0.673 **	−0.555 **	−0.530**	−0.356	0.249	−0.040	−0.311	−0.407 *
		8000 Hz	−0.422 *	−0.574 **	−0.468 *	−0.313	−0.139	0.038	−0.467 *	−0.437 *
	Speech With Noise	0 dB SNR	−0.479 *	−0.213	0.350	0.331	0.482 *	−0.172	−0.481 *	−0.190
		−6 dB SNR	−0.224	−0.105	−0.078	−0.325	−0.340	−0.275	−0.156	−0.208
YG	Tone	250 Hz	−0.381	−0.183	0.127	−0.086	0.041	−0.471 *	0.092	−0.412 *
		500 Hz	−0.415 *	−0.363	−0.025	−0.045	−0.160	−0.333	−0.245	0.045
		4000 Hz	−0.290	−0.045	−0.025	−0.129	0.038	−0.087	−0.320	−0.497 *
		8000 Hz	−0.311	−0.311	−0.042	−0.065	−0.042	−0.097	−0.269	−0.363
	Speech With Noise	0 dB SNR	−0.141	−0.229	−0.058	−0.086	−0.164	0.056	0.104	−0.384
		−6 dB SNR	−0.273	−0.578 **	−0.297	−0.383	−0.633 **	−0.326	−0.293	−0.410 *

Abbreviations: OG, older adults; YG, young adults; SNR, signal-to-noise ratio; *, *p* < 0.05; **, *p* < 0.01.

**Table 4:** Summary of the multiple regression analysis for listening effort and tone detection task for both groups.

Group		B	*SE*	*β*	*t*	*p*
OG	Intercept	3.842	0.520		7.384	0.000
	0° at 4000 Hz	−0.030	0.004	−0.684	−6.791	0.000
	250 Hz of HT	−0.088	0.016	−0.516	−5.431	0.000
	1000 Hz of HT	0.059	0.011	0.487	5.253	0.000
	0° at 500 Hz	0.014	0.005	0.285	2.709	0.014
	Summary	R^2^ = 0.928, Adjusted R^2^ = 0.826, F = 7.304, *p* = 0.014				
		B	SE	*β*	*t*	*p*
YG	Intercept	5.348	0.981		5.454	0.000
	315° at 4000 Hz	−0.028	0.010	−0.497	−2.746	0.012
	Summary	R^2^ = 0.497, Adjusted R^2^ = 0.214, F = 7.542, *p* = 0.012				

Abbreviations: OG, older adults; YG, young adults; SE, standard error; HT, hearing threshold; SSQ, the speech, spatial, and qualities of hearing scale.

**Table 5:** Summary of the multiple regression analysis for listening effort and speech segregation from background noise tasks (0 dB and −6 dB SNR) in two groups.

OG	0 dB SNR		**B**	**SE**	***β***	***t***	***p***
		Intercept	5.690	1.307		4.353	0.000
		180°	0.036	0.014	0.402	2.597	0.017
		0°	−0.033	0.010	−0.507	−3.150	0.005
		Summary	R^2^ = 0.714, Adjusted R^2^ = 0.440, F = 4.796, *p* = 0.040				
	−6 dB SNR		**B**	**SE**	***Β***	***t***	***p***
		Intercept	6.907	0.279		24.748	0.000
		250 Hz of HT	0.036	0.011	0.549	3.192	0.004
		135°	−0.012	0.004	−0.461	−2.681	0.014
		Summary	R^2^ = 0.624, Adjusted R^2^ = 0.333, F = 7.186, *p* = 0.014				
YG	0 dB SNR		**B**	**SE**	***β***	***t***	***p***
		Intercept	4.084	0.219		18.661	0.000
		3000 Hz of HT	−0.110	0.049	−0.423	−2.237	0.035
		Summary	R^2^ = 0.423, Adjusted R^2^ = 0.143, F = 5.005, *p* = 0.035				
	−6 dB SNR		**B**	**SE**	***Β***	***t***	***p***
		Intercept	11.261	1.051		10.718	0.000
		180°	−0.046	0.013	−0.552	−3.514	0.002
		3000 Hz of HT	−0.107	0.042	−0.354	−2.518	0.020
		45°	−0.029	0.012	−0.380	−2.479	0.022
		Summary	R^2^ = 0.785, Adjusted R^2^ = 0.562, F = 6.145, *p* = 0.022				

Abbreviations: SE, standard error; SNR, signal-to-noise ratio; HT, hearing threshold; SSQ, the speech, spatial, and qualities of hearing scale.

**Figure A1:**
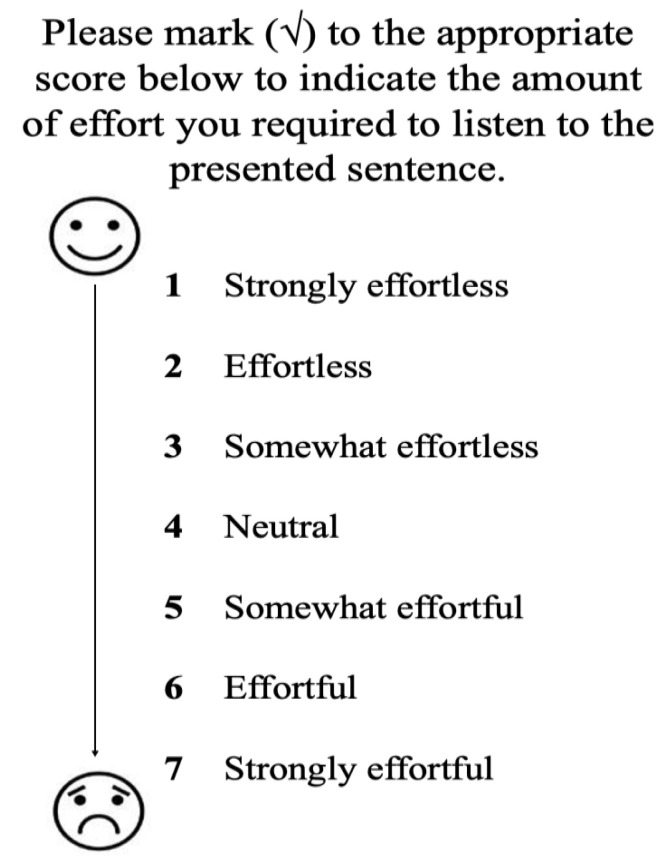
An example of the seven-point Likert scale questionnaire used for evaluating amount of listening effort.

## Data Availability

Not applicable.
